# Exploring the role of wastewater-based epidemiology in understanding tuberculosis burdens in Africa

**DOI:** 10.1016/j.envres.2023.115911

**Published:** 2023-08-15

**Authors:** Hlengiwe N. Mtetwa, Isaac D. Amoah, Sheena Kumari, Faizal Bux, Poovendhree Reddy

**Affiliations:** a Institute for Water and Wastewater Technology (IWWT), Durban University of Technology, PO Box 1334, Durban, 4000, South Africa; b Department of Community Health Studies, Faculty of Health Sciences, Durban University of Technology, PO Box 1334, Durban, 4000, South Africa; c Department of Environmental Science, University of Arizona, Tuscon, USA

**Keywords:** Tuberculosis, Wastewater-based epidemiology, *Mycobacterium tuberculosis* complex, Sub-saharan Africa

## Abstract

Tuberculosis (TB) remains a persistent challenge to public health and presents a substantial menace, especially in developing nations of sub-Saharan Africa. It exerts a considerable strain on healthcare systems in these regions. Effective control requires reliable surveillance, which can be improved by incorporating environmental data alongside clinical data. Molecular advances have led to the development of alternative surveillance methods, such as wastewater-based epidemiology. This studyinvestigated the presence, concentration, and diversity of *Mycobacterium tuberculosis* complex, the cause of TB, in from six African countries: Ghana, Nigeria, Kenya, Uganda, Cameroon, and South Africa. Samples were collected from wastewater treatment plants. All samples were found to contain *Mycobacterium* species that have been linked to TB in both humans and animals, including *Mycobacterium tuberculosis* complex, *Mycobacterium tuberculosis*, *Mycobacterium bovis*, *Mycobacterium africanum*, and *Mycobacterium caprae*, at varying concentrations. The highest median concentration was found in Ghana, reaching up to 4.7 Log copies/ml for MTBC, 4.6 Log copies/ml for *M. bovis*, and 3.4 Log copies/ml for *M. africanum*. The presence of *M. africanum* outside of West Africa was found in South Africa, Kenya, and Uganda and could indicate the spread of the pathogen. The study underscores the usefulness of wastewater-based epidemiology for tracking TB and shows that even treated wastewater may contain these pathogens, posing potential public health risks.

## Highlights

• Sub-Saharan Africa faces TB challenge; wastewater data may improve surveillance.
• Wastewater-based epidemiology approach detects MTBC species in African countries.
• Ghana had the highest median concentration of TB pathogens in wastewater.
• Treated wastewater may harbour TB pathogens, posing public health risks.

## 1 Introduction

Tuberculosis (TB) continues to pose a substantial public health burden globally, placing significant pressure on healthcare systems, particularly in developing countries, particularly those in Sub-Saharan Africa (Ameke et al., 2021). TB is becoming increasingly untreatable as multidrug-resistant strains emerge globally and the burden of human immunodeficiency virus (HIV)/TB coinfection rises (Ameke et al., 2021). Weak healthcare systems, the HIV epidemic, societal conditions, the growth of multidrug-resistant TB (MDR-TB), and a shortage of laboratories make the situation worse, especially in Africa, where the incidence rate of TB is over 25% (Gandhi et al., 2010). South Africa, Angola, Ethiopia, Ghana, Uganda, Kenya, and the Democratic Republic of the Congo had the highest TB-HIV burdens in Africa in 2017 (Assob et al., 2017; WHO, 2019).

In addition to human tuberculosis, bovine tuberculosis caused by *Mycobacterium bovis* is a significant economic and public health concern in cattle. The consumption of unpasteurized milk is believed to be the primary means of exposure to zoonotic TB in humans in developing countries, as stated by Macedo Couto et al. (2019) and Adesokan et al. (2019). Those who frequently interact with animals, such as pastoralists and abattoir workers, are at a heightened risk of developing pulmonary TB from inhaling aerosols released by infected animals. Humans infected with active TB, including farm workers, are the primary source of *M. tuberculosis* infection and disease in animals, including cattle, due to environmental contamination resulting from the release of cough aerosol, infected urine, faeces, or sputum. This has been supported by studies conducted by Donald et al. (2018), Randall et al. (2021), Adesokan et al. (2019), Kanipe and Palmer (2020). According to estimates from the World Health Organization (WHO), zoonotic TB caused 12,500 fatalities and 142,000 new cases in 2017 (WHO, 2018). However, due to inconsistent surveillance data from most countries, these figures are likely to be underestimated (Zimpel et al., 2020). In the surveillance of tuberculosis infections, clinical diagnoses and hospitalization data have been extensively used (Migliori et al., 2021). The reliance on clinical case reports, hospital admissions and clinical surveys, as surveillance methods, has proven to be a challenge, particularly where there are other competing interests for scarce resources (Iskandar et al., 2021). Some of these challenges include resource constraints, high costs involved in maintaining these surveillance tools and potential reporting biases (Jayatilleke, 2020). To achieve the United Nations' Sustainable Development Goals of eradicating TB by 2030, future strategies for prevention and control need to address all forms of TB in humans, including its interaction with animals, and prioritize the establishment of reliable and comprehensive surveillance systems (Zimpel et al., 2020).

Improved surveillance systems may assist authorities to respond effectively in developing policies for managing and controlling diseases. Therefore, the development and implementation of alternative surveillance tools for identifying disease severity and the emergence of novel strain and resistance patterns is a top priority. One such strategy is the use of sewage or wastewater-based analysis, commonly referred to as wastewater-based epidemiology (WBE). This approach has received attention lately due to its role in developing early warning and surveillance of COVID-19 infections (Pillay et al., 2021; Bivins et al., 2020, Zhu et al., 2021) and the surveillance of polio (Brouwer et al., 2018). The detection of these infectious bacilli for TB in treated wastewater could provide insight into the possible risks of TB infections through the environmental route. There is an increasing body of evidence to indicate that water could be an important vehicle for the transmission of these pathogens (Dufour, 2004; WHO, 2011; Sims and Kasprzyk-Hordern, 2020). This paper presents the use of molecular approaches and WBE for the surveillance of TB in sub-Saharan African countries from both untreated and treated wastewater.

## 2 Materials and methods

### 2.1 Study location

Wastewater samples were collected from six African countries for this study (Ghana, Nigeria, Kenya, South Africa, Uganda, and Cameroon). These countries represent West (Ghana and Nigeria), Central (Cameroon), East (Uganda and Kenya), and Southern Africa (South Africa), providing a representation of sub-Saharan Africa. In each country, one WWTP was selected, except South Africa where three WWTPs were sampled due to ease of access and logistical support, however, data from these WWTPs was normalized to represent the country and not each WWTP. The WWTPs were selected based on their capacity to serve at least 5000 people and the treatment of hospital sewage (Table 1).

**Table 1:** Information on the wastewater treatment facilities used in this study.

WWTP	City	Design Capacity (Mℓ/d)	Remarks
South African- WWTP A	Durban	18.8	The hospital, which has 17 affiliated clinics, offers healthcare services to the community at both regional and district levels, and receives support from them
South African- WWTP B	Durban	4.90	Receives from a hospital that serves as both a receiving and a referring facility for other hospitals and clinics.
South African- WWTP C	Durban	70.0	Obtains specialized care for complex cases of tuberculosis (TB) and multidrug-resistant (MDR) TB from a hospital complex dedicated to providing these services
Ghana	Kumasi	0.22	Receiving domestic wastewater from hospitals and major animal farms including small scale poultry farms and waste from abattoir
Nigeria	Ibadan	0.817	Receives wastewater from a teaching hospital
Kenya	Kisumu	7.95	Received input from two hospitals, no input from pharmaceutical industries and no input from major animal farms
Uganda	Kampala	45	Receives input from hospitals. No input from pharmaceutical industries, although, there are a number of businesses dealing in pharmaceutical products that are connected to the sewer lines. No input from major animal farms
Cameroon	Yaoundé	0.801	Domestic sewage from social housing is received, but none from hospitals, livestock farms, or the pharmaceutical industry

The research by Mtetwa et al. (2022) and Mtetwa et al. provided data for South Africa (2022). Collaborators from other chosen African nations contributed information for their countries.

### 2.2 Sample collection, preparation and processing

At each WWTP, a one-time composite sample of 1 L was taken from the treated wastewater (post-chlorinated effluent) and untreated wastewater (influent). As a result, per WWTP, two 1-L samples of influent and effluent were collected. Numerous subsamples (100 ml) were collected at 30-s intervals until the 1-L composite samples were obtained. A commercial courier was used to transport samples from the five African nations to the Institute for Water and Wastewater Technology laboratories in Durban, South Africa, where they were analyzed within 48 h. Prior to analysis, homogenization of samples was performed, and 50 mL subsamples were collected. These subsamples were then centrifuged at 3000 revolutions per minute for 20 min, followed by removal of the supernatant. The DNeasy Powersoil DNA extraction kit was used to recover total DNA from the resultant pellet, in accordance with the manufacturer's instructions (supplied by QIAGEN, Germany). The extracted DNA was evaluated for quantity and quality using the NanoPhotometer (NP80—All-in-One Spectrophotometer, provided by IMPLEN). Each analysis was replicated three times to ensure precision and accuracy.

### 2.3 Concentrations of *Mycobacterium* spp. in untreated and treated wastewater

An optimized droplet digital PCR (ddPCR) method, as detailed by Mtetwa et al. (2022), was utilized for the detection of *M. tuberculosis*. The 20 μL reaction mixture included 10 μL of 2X QX200 ddPCR EvaGreen Supermix, template DNA quantified using an IMPLEN Nano-Photometer NP80 at concentrations ranging from 1 to 20 ng/μL, forward and reverse primers each at a final concentration of 250 nM, and RNase/DNase-free water. Droplets were produced using an automated droplet generator and the reaction was subjected to thermal cycling, which involved an initial denaturation step at 95 °C for 10 min, followed by 30 cycles of 96 °C for 45 s with varying annealing temperatures for each primer and organism. The ddPCR plates were analyzed using the QX200 droplet reader and QuantaSoft™ analysis Pro software was used to analyze the droplet counts and amplitudes. The limit of detection (LOD) was determined as 3.0 (±0.06) Log copies/ml by ten-fold serial dilutions.

### 2.4 Statistical analysis

The data was recorded in Microsoft Excel (USA), and the normality of the data was assessed using the Akaike Information Criterion (AIC) calculated with @Risk (Palisade Inc. USA). Based on the normality tests, the Kruskal-Wallis tests were applied to compare the concentrations of different *Mycobacterium* species that cause tuberculosis, followed by Dunn's Multiple Comparison tests. A 95% confidence interval was applied to all statistical tests, and a p-value less than 0.05 was considered statistically significant. The statistical analyses were performed using GraphPad Prism (Version 9.0, GraphPad Software, USA).

## 3 Results

### 3.1 Concentrations tuberculosis related *Mycobacterium* spp. in untreated wastewater

All of the samples contained total mycobacteria, which refers to organisms in the genus *Mycobacterium*, as detected by the 16s rRNA gene. The highest median concentration of total mycobacteria was observed in Ghana at 4.8 (±0.73) Log copies/ml, and the lowest was in Cameroon at 4.2 (±0.82) Log copies/ml. The results of a Kruskal-Wallis test showed a significant difference among the countries (p ≤ 0.05), driven by the differences in concentrations between Uganda and Cameroon. The second-highest concentrations were found in the *Mycobacterium tuberculosis* complex (MTBC), which consists of *M. tuberculosis*, *M. bovis*, *M. caprae*, and *M. africanum*. The MTBC concentrations followed a similar trend as total mycobacteria, with the highest median concentrations in Ghana at 4.7 (±0.87) Log copies/ml and the lowest in Cameroon at 3.4 (±0.94) Log copies/ml. Multiple comparison tests showed no significant differences between countries except for Ghana and Cameroon, which showed a statistically significant difference (p ≤ 0.05). At the species level, the most abundant species of MTBC was *M. bovis*, with high median concentrations of 4.6 (±0.86) Log copies/ml in Ghana and the lowest concentrations in Uganda and Cameroon. The main causative agent of human TB, *M. tuberculosis*, was the third most abundant species of MTBC, with the highest median concentration of 3.9 (±0.17) Log copies/ml observed in Uganda and closely followed by South Africa at 3.5 (±0.048) Log copies/ml. *M. africanum* had the highest median concentrations in Ghana at 3.8 (±0.01) Log copies/ml, in Kenya at 3.4 (±0.79) Log copies/ml, and in South Africa at 3.2 (±0.24) Log copies/ml, and the lowest in Cameroon at 2.5 (±1.63) Log copies/ml. *M. caprae* had the highest median concentrations of 3.8 ± 0.01 Log copies/ml in South Africa and the least in Ghana (2.1 ± 1.95 Log copies/ml).

**Fig. 1:**
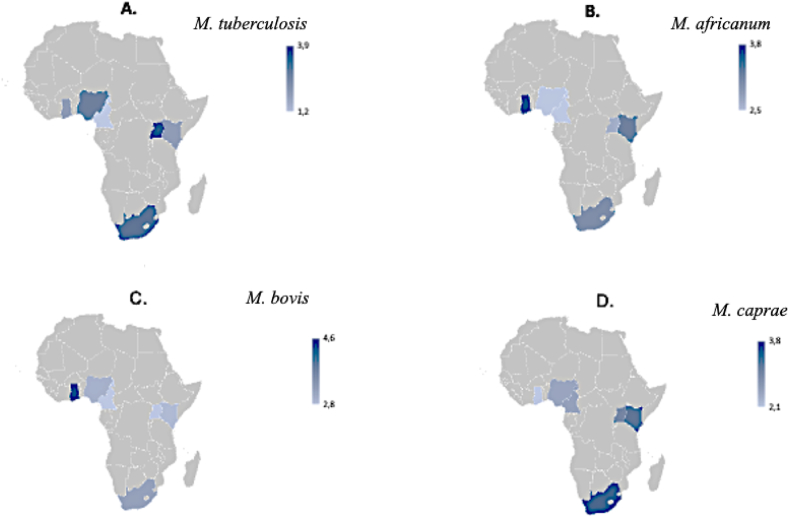
Graphical representation of the relative concentration levels of *M. tuberculosis* (A), *M. africanum* (B), *M. bovis* (C), and *M. caprae* (D) found in untreated wastewater samples collected from six sub-Saharan African countries.

The concentrations of the four species, *M. tuberculosis*, *M. africanum*, *M. bovis*, and *M. caprae*, in untreated wastewater varied among the six countries (p < 0.05), but no significant differences between countries were indicated by Dunn's multiple comparison tests, except for specific comparisons between Uganda and Cameroon for *M. tuberculosis*, Cameroon and South Africa for *M. africanum*, Ghana and Uganda, and Ghana and South Africa for *M. bovis*, and between Ghana and South Africa for *M. caprae* (Fig. 1).

### 3.2 In-country variation in concentration of tuberculosis-causing *mycobacterium* species in untreated wastewater

In Ghana, the highest median concentration of *Mycobacterium* species that cause tuberculosis was found in *M. bovis*, with a concentration of 4.6 ± 0.86 Log copies/ml. In contrast, the lowest median concentration was recorded in *M. caprae,* at 2.1 (±1.95) Log copies/ml. Although multiple comparisons tests did not reveal significant differences between the species, there were significant differences observed between total mycobacteria and *M. tuberculosis*, as well as between total mycobacteria and *M. africanum.* Similarly, In Cameroon the highest median concentration was observed for *M. bovis,* at 2.8 (±0.10) Log copies/ml, and the lowest in *M. tuberculosis*, at 1.2 (±0.74) Log copies/ml. No significant differences were observed between these species, but there was a significant difference between total mycobacteria and *M. tuberculosis*.

In Nigeria, *M. tuberculosis* and *M. bovis* had the highest median concentrations, both at 3.1 (±0.46) and 3.1 (±0.23) Log copies/ml, respectively, while the lowest median concentrations were found in *M. africanum* and *M. caprae*, at 2.6 (±1.35) and 2.5 (±0.75) Log copies/ml, respectively. No significant differences were seen between these species. In Uganda, *M. tuberculosis* was the most abundant species with a concentration of 3.9 (±0.17) Log copies/ml, while the least abundant were *M. africanum* and *M. bovis*, at 2.8 Log copies/ml. All species showed a statistically significant difference in median concentration in Uganda.

In contrast, the most abundant species detected in Kenya was *M. caprae,* followed by *M. africanum,* and the lowest was *M. tuberculosis,* at 2.2 (±1.85) Log copies/ml. All species showed a statistically significant difference in concentration in Kenya, except for total mycobacteria and *M. tuberculosis*. However, in South Africa, all four species, *M. caprae, M. tuberculosis, M. bovis,* and *M. africanum,* had similar concentrations, ranging from 3.8 (±0.10) to 3.2 (±0.24) Log copies/ml. No significant differences were found between the species, but there were significant differences observed between total mycobacteria and *M. bovis.*

**Fig. 2:**
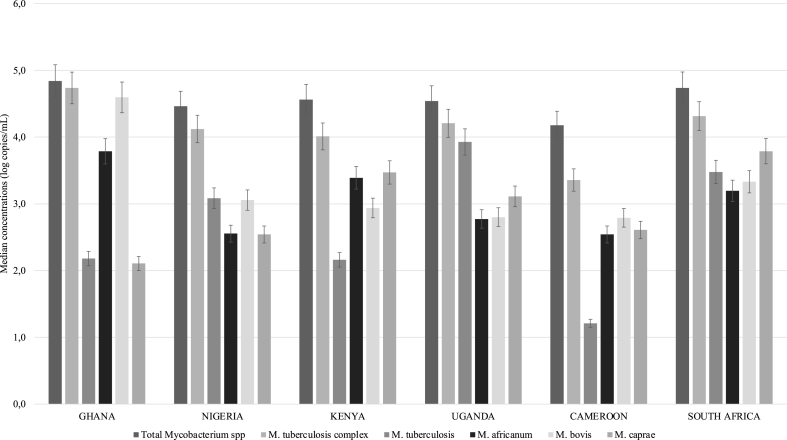
Median concentrations of tuberculosis-causing *Mycobacterium* spp. in untreated wastewater from the six African countries.

The results show that in some countries (e.g. Uganda), there were significant differences in the concentration of different species, while in others (e.g. South Africa), the concentrations of all species were similar and no significant differences were found (Fig. 2).

#### 3.2.1 The concentrations of tuberculosis-causing *Mycobacterium* spp. in treated wastewater

**Fig. 3:**
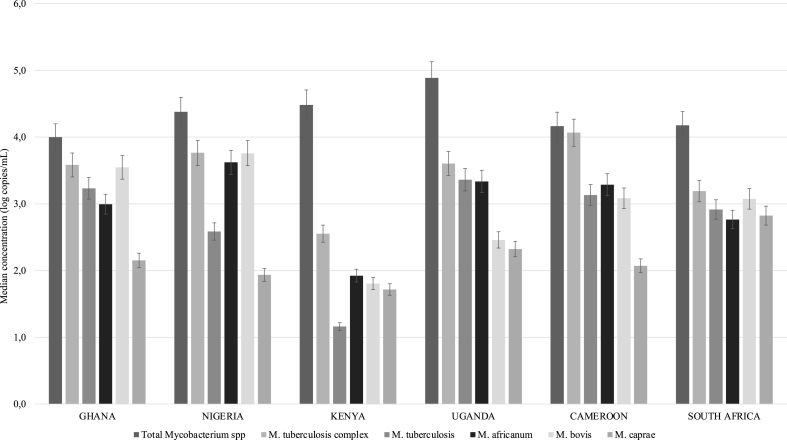
Median concentrations of tuberculosis-causing *Mycobacterium* spp. in treated wastewater from the six African countries.

Total *Mycobacterium* spp. in the treated wastewater from six countries, had median concentrations ranging from 4.0 to 4.9 Log copies/ml. The highest concentrations were found in Uganda [4.9 (±0.55) Log copies/ml]. The Kruskal-Wallis test indicated a significant difference in the total mycobacteria concentrations among the countries (p ≤ 0.05), largely due to significant difference between Uganda and Nigeria, as indicated by Dunn's multiple comparison tests. *M. tuberculosis* complex was the second most abundant after total mycobacteria, with median concentrations ranging from 2.6 to 4.4 Log copies/ml. These are expected results, as MTBC can be considered a subset of total mycobacteria. The highest concentration of this *M. tuberculosis* complex was observed in Cameroon. No significant difference was observed between all the countries, except for Nigeria and Uganda, with a statistically significant difference (p = 0.0087). The analysis of *M. tuberculosis* in treated wastewater showed the highest median concentrations in Uganda (3.6 (±0.09) Log copies/ml) and the least in Kenya (1.2 (±0.18) Log copies/ml). No statistically significant difference was observed among all the countries, except between Kenya and Uganda (p ≤ 0.05) (Fig. 3). *M. africanum* was most abundant in Nigeria and least in Kenya, while *M. bovis* showed a similar trend, with the highest median concentration in Nigeria and the lowest in Kenya (Fig. 3). However, this difference was not statistically significant. The highest median concentration for *M. caprae* was observed in South Africa and a significant variation across the selected countries was indicated by the Kruskal-Wallis test, with no statistically significant variation except for Kenya and South Africa.

#### 3.2.2 In-country variation in concentration of tuberculosis-causing *mycobacterium* species in treated wastewater

An intra-country disparity in the concentrations of these organisms was detected. For instance, in Ghana, *M. bovis* was the most abundant of the four MTBC species, while *M. caprae* was the least abundant with a concentration of 2.2 (±1.57) Log copies/ml. The differences in median concentrations among the species in Ghana were statistically significant (p ≤ 0.05), attributed to differences in the concentration of total mycobacteria and *M. caprae*. Treated wastewater from Nigeria and South Africa showed similar variation in the concentrations of the selected organisms, as observed in Ghana, but with a statistically significant difference in the mean concentrations of the group and species detected in Nigeria. In Kenya, *M. africanum* was the most abundant tuberculosis-causing bacteria in treated wastewater (1.9 (±0.75) Log copies/ml), while *M. tuberculosis* was the least abundant. In Uganda, human-causing tuberculosis species (*M. tuberculosis* and *M. africanum*) were more abundant than animal-causing species (*M. bovis* and *M. caprae*). In Cameroon, *M. africanum* was the most abundant species detected [3.3 (±0.61) Log copies/ml] and *M. caprae* was the least abundant, besides the high concentration of total mycobacteria and *M. tuberculosis* complex. The median concentrations varied significantly among all analyzed organisms, with a significant difference found between total mycobacteria and *M. caprae*, and between *M. tuberculosis* complex and *M. caprae* according to the Dunn's multiple comparisons test.

## 4 Discussion

This study found varying abundances of different *Mycobacterium* species in wastewater from six sub-Saharan African countries. Total mycobacteria in the untreated wastewater observed in all the countries represent all the species of the *Mycobacterium* genus including non-tuberculous mycobacteria which may explain the abundance in selected African countries. These, also known as environmental mycobacteria, comprise more than 150 species and are found worldwide in both natural and man-made environments (Nishiuchi et al., 2017). According to genomic studies, all MTBC species exhibit between 95% and 100% DNA relatedness, having the identical 16 S rRNA gene (Coll et al., 2014; Zumla et al., 2017). The wastewater samples collected for this study were mainly domestic sewage, hospital sewage, slaughterhouses and crops and animal farms. The highest abundance of total *Mycobacterium* species and *M. tuberculosis* complex, causative agents of tuberculosis in both humans and animals were observed in Ghana. This may be due to the sources of sewage received by this WWTP (Table 1), which are hospitals, major animal farms including small-scale poultry farms and waste from an abattoir. These different sources are known to harbour different types of mycobacteria, therefore could have played a significant role in the concentration of total mycobacteria and *M. tuberculosis* complex in untreated wastewater in Ghana.

TB may be caused by *M. tuberculosis* or *M. africanum* (mainly in West Africa). The concentration of these two pathogens in the wastewater indicates the main driver of TB infections in the respective countries. For example, in countries such as Ghana, Cameroon and Kenya, the concentrations of *M. africanum* were higher than *M. tuberculosis*, which perhaps could be attributed to the higher infections caused by the former. This is expected for countries within the West Africa subregion, such as Ghana and to a lesser extent Cameroon (Gehre et al., 2016). However, the detection of high concentrations of *M. africanum* (3.4(±0.79) Log copies/ml) in Kenya, compared to *M. tuberculosis* (2.2(±1.85) Log copies/ml) is surprising, considering that Kenya is located within East Africa. Furthermore, Nigeria, one of the most populous nations in Africa with high TB incidence had lower concentrations of *M. africanum* (2.6 (±1.35) Log copies/ml) compared to *M. tuberculosis* (3.1 (±0.46) Log copies/ml). This again contradicts the expectation that West African countries would have comparable *M. africanum* and *M. tuberculosis* related infections. Nigeria is ranked seventh among the 30 countries with a high TB burden, and second to South Africa in Africa (Kanabus, 2022). While *M. tuberculosis* is found worldwide, *M. africanum* is commonly found in West Africa, where it accounts for up to 50% of TB cases (Asare et al., 2018). It has also been identified in other countries outside West Africa such as Germany, the United Kingdom, France, Spain, the United States of America and Kazakhstan (Rodriguez-Campos et al., 2014). An 8-year cohort study by Asare et al. (2018) in Ghana found that the prevalence of *M. africanum* is fairly constant at approximately 20%, indicating that *M. africanum* and *M. tuberculosis* may be transmitted equally. The high abundance of *M. africanum* in Cameroon as compared to *M. tuberculosis* could be attributed to various factors such as migration from the western part to the central part of Africa including Cameroon (Noeske et al., 2016; Chihota et al., 2018). Given the prevalence of the *M. africanum* strain in Nigeria, a neighbouring country to Cameroon and the Central African Republic, both of which have significant TB burdens, this could be one of several reasons for the abundance of *M. africanum* in Cameroon (Koro Koro et al., 2016). While there have been occasional reports of the isolation of this pathogen from primates and bovines in Gabon, Liberia, and Sierra Leone, such findings have not been systematically validated (Rodriguez-Campos et al., 2014). A possible explanation for the elevated incidence of *M. africanum* is its ability to stably adapt to certain human populations (Ameke et al., 2021). Additionally, the close evolutionary affinity between *M. africanum* and animal isolates raises the possibility of a zoonotic origin for this bacterium. A noteworthy discovery from this research is the observation of comparable levels of *M. tuberculosis* (3.5 ± 0.048 Log copies/ml) and *M. africanum* (3.2 ± 0.24 Log copies/ml) in South African wastewater. The implication of this is that *M. africanum* may have a greater role in tuberculosis transmission than previously assumed. The identification of these microorganisms may be linked to the movement of individuals from West Africa to other regions in Africa, which could potentially facilitate the dissemination of *M. africanum* mycobacteria responsible for causing tuberculosis among the population (Dhavan et al., 2017).

The high abundance of tuberculosis-causing *Mycobacterium* species commonly reported in animals (*M. bovis* and *M. caprae*) is noteworthy. This could be a result of humans being carriers of these species (El-Sayed et al., 2016) due to ingestion of contaminated milk, and exposure to infected animals (Prodinger et al., 2014). Furthermore, the incidence of zoonotic bovine tuberculosis (BTB) may be linked to subsistence farming in adjacent populations and inadequate monitoring for BTB in domestic animals. The close contact between humans and livestock during herd management could also facilitate the emergence and dissemination of zoonotic BTB under favourable circumstances (Tenguria et al., 2011; Awah-Ndukum et al., 2019).

In locations where the wastewater collected did not come from animal farms or slaughterhouses, high concentrations of *M. bovis* and *M. caprae* were discovered. Ghana, for instance, exhibited the highest concentration of *M. bovis* at 4.6 (±0.86) Log copies/ml. Yeboah-Manu et al., 2016 reported that animal strains were detected in some patients who did not have frequent direct contact with cattle or other animal farms. This finding is in contrast to a study conducted in Mexico, where the majority of patients from whom MTBC animal strains were recovered had not had direct contact with animals but had consumed unpasteurized milk products. Previous studies have shown that environmental contamination from faecal shedding can provide potential and indirect routes for the transmission of *M. bovis* infections (Travis et al., 2019; Wu, 2020). *M. bovis* cells have been detected in oro-nasal mucus, sputum, urine, faeces, and wound discharges in many animals (Corner et al., 2012; Barasona et al., 2017; Barbier et al., 2017; Vayr et al., 2018). In Kampala, Wanzala et al. (2019) reported that although *M. tuberculosis* is the most common *M. tuberculosis* complex infection, infections with *M. tuberculosis* and *M. bovis* are clinically and pathologically indistinguishable, which could account for the prevalence of pulmonary tuberculosis in the country.

One other major finding worth discussing is that human-adapted *M. tuberculosis* complex species were less abundant in Kenya compared to the animal-adapted species. *M. africanum* (human-adapted) and *M. caprae* (animal-adapted) were the most abundant species in comparison to *M. bovis* in the untreated wastewater from Kenya. The simultaneous presence of *M. caprae* and *M. bovis* is significant since these two agents are the principal etiological agents of zoonotic tuberculosis. While *M. caprae* accounts for only a small fraction of the overall burden of human tuberculosis, it infects a considerable number of domestic and wild ungulates and is viewed as a condition with public health implications (Paul, 2021).

The movement of livestock may also contribute to the spread of disease across neighbouring countries. Livestock movements in Africa are mostly driven by the necessity for animals to gain access to resources (such as grazing and watering) to secure their survival. Livestock frequently travels several kilometres each day to reach shared resource regions, where extensive herd mixing and animal contact occurs, posing significant risks of pathogen transmission and disease spread to other areas (Ekwem et al., 2021).

High concentrations of tuberculosis-causing *Mycobacterium* species were observed in treated wastewater from the six selected African countries. In countries such as Kenya and Cameroon, concentrations in the treated wastewater were higher than in the untreated wastewater. The concentrations in the treated wastewater indicate the contribution of these WWTPs to their occurrence in the receiving water environment. The presence of these organisms in treated wastewater raises concern about risks related to exposure (directly or indirectly) to this wastewater or surface water contaminated with this wastewater. The high concentrations observed may also be due to their resistance to environmental conditions. Several factors, including moisture, salt-tolerant, temperature, inhibitors, pH and solar radiation protection, have been reported to influence MTBC survival in various matrices (Mtetwa et al., 2022; Barbier et al., 2017; Asmar et al., 2016). Tuberculosis-causing bacteria are amoeba-resistant, which may help them survive in the environment, particularly in wastewater (Ghodbane et al., 2014; Drancourt, 2014). *M. tuberculosis* and *M. bovis* could persist in amoebal trophozoites for hours to days (Sanchez-Hidalgo et al., 2017; Butler et al., 2020; Fellag et al., 2021). Some studies have challenged *M. tuberculosis* as an obligate pathogen with no environmental niche (Minnikin et al., 2015; Aboubaker Osman et al., 2016). The argument is that these strains could have been opportunist mycobacteria that could survive in the hostile environment of an animal stomach and other possible matrices, rather than obligatory pathogens (Aboubaker Osman et al., 2016; Fellag et al., 2021). This supports the abundance of these organisms in environmental matrices and suggests that the sources of environmental *M. tuberculosis* could be both animals and humans (Kanipe and Palmer, 2020; Romha et al., 2018; Brites et al., 2018). It's hardly unexpected that *M. tuberculosis*, an organism that developed from a soil saprophyte and is comparatively resilient to environmental stresses due to its capsule, mycolic acid, and lipid-rich cell wall, would be capable of surviving for extended periods outside of its host; as in the wastewater treatment plants (Martinez et al., 2019).

### 4.1 Limitations of this study

• Representativeness: The study may not accurately represent the overall TB burden in the population as it only uses wastewater samples from certain areas and not from the entire population.
• Technical limitations (a possibility but highly unlikely with the method used in this study): The accuracy of the results may be influenced by the technical limitations of the wastewater-based epidemiology method, such as the presence of interfering substances in the samples, the accuracy of the detection methods, and the possibility of false-positive results.
• Sample size: The sample size used in the study may be too small to accurately estimate the TB burden in the population, leading to potential inaccuracies in the results. However, this data is to highlight the possible detection of these MTBC species in wastewater from these countries, which with longitudinal studies can provide accurate information on the occurrence and distribution of these species in relation to the available clinical data in that particular area.
• Generalizability: The findings of the study may not be generalizable to other populations or countries as the TB burden, wastewater systems, and treatment processes can vary greatly between locations. However, the WBE approach can be adopted in any country using suitable methods for their conditions.
• Environmental factors: The study did not account for environmental factors that can impact the accuracy of the results, such as temperature, rainfall, and the presence of other infectious agents in the wastewater.

These limitations should be considered when interpreting the results and applying the findings of the study to other populations or settings.

## 5 Conclusion and recommendations

This study aimed to assess the presence, concentration, and diversity of tuberculosis-causing *Mycobacterium tuberculosis* complex (MTBC) in wastewater from six sub-Saharan African countries (Ghana, Nigeria, Kenya, Uganda, Cameroon, and South Africa). The results showed that all the wastewater samples contained known MTBC species at varying concentrations, with the highest median concentrations detected in untreated wastewater from Ghana. The study also revealed that the concentrations of MTBC species in treated wastewater varied significantly among the countries and that *M. africanum* was most abundant in Ghana but also detected in other countries outside of West Africa, which could be an indication of infections within these populations. The results of this study highlight the utility of wastewater-based epidemiology in tracking tuberculosis infections and emphasize the dissemination of these pathogens in the environment, which could have an adverse public health impact on downstream users. This multi-country application of wastewater-based epidemiology in Africa provides valuable information for tuberculosis control efforts and highlights the importance of including environmental data in disease surveillance. This study highlights the importance of the One Health approach in addressing global public health issues like TB. The interconnectedness of human, animal, and environmental health means that solutions must be multi-disciplinary and integrated. The findings also have implications for the Sustainable Development Goals (SDGs), particularly SDG 3 (Good Health and Well-being) and SDG 6 (Clean Water and Sanitation), as they demonstrate the importance of addressing TB and promoting access to clean water and sanitation to achieve a healthier, more sustainable world.

## Author contributions

Category 1

Conception and design of study: Hlengiwe Nombuso Mtetwa; Isaac Dennis Amoah; Sheena Kumari; Poovendhree Reddy; Faizal Bux; acquisition of data: Hlengiwe Nombuso Mtetwa, analysis and/or interpretation of data: Hlengiwe Nombuso Mtetwa, Isaac Dennis Amoah

Category 2

Drafting the manuscript: Hlengiwe Nombuso Mtetwa, revising the manuscript critically for important intellectual content: Hlengiwe Nombuso Mtetwa, Isaac Dennis Amoah.

Category 3

Approval of the version of the manuscript to be published (the names of all authors must be listed): Isaac Dennis Amoah; Sheena Kumari; Poovendhree Reddy; Faizal Bux; Hlengiwe Nombuso Mtetwa.

## Declaration of competing interest

The authors declare that they have no known competing financial interests or personal relationships that could have appeared to influence the work reported in this paper.

## Data Availability

Data will be made available on request.
