# Supplementary material for: Exploring the role of wastewater-based epidemiology in understanding tuberculosis burdens in Africa
Source: Environ Res. 2023 Aug 15;231:115911. doi: 10.1016/j.envres.2023.115911 (PMC10318412; doi:10.1016/j.envres.2023.115911)
Supplement: Multimedia component 1 [file mmc1.docx]

Supplementary data

Table S1: Concentrations of *Mycobacterium* species related to tuberculosis infections.

|  | Total mycobacteria |  | *M. tuberculosis* complex |  | *M. tuberculosis* |  |
| --- | --- | --- | --- | --- | --- | --- |
|  | Influent | Effluent | Influent | Effluent | Influent | Effluent |
|  | Median (±SD) | Median (±SD) | Median (±SD) | Median (±SD) | Median (±SD) | Median (±SD) |
| Ghana | 4.8 (±0.73) | 4.0 (±1.86) | 4.7 (±0.87) | 3.6 (±0.41) | 2.2 (±1.79) | 3.2 (±0.75) |
| Nigeria | 4.5 (±0.37) | 3.6 (±0.81) | 4.1 (±0.17) | 4.4 (±0.24) | 3.1 (±0.46) | 2.6 (±0.35) |
| Kenya | 4.6 (±-.17) | 4.5 (±0.77) | 4.0 (±0.86) | 2.6 (±0.73) | 2.2 (±1.85) | 1.2 (±0.18) |
| Uganda | 4.5 (±0.95) | 4.9 (±0.55) | 4.2 (±0.07) | 2.5 (±1.64) | 3.9 (±0.17) | 3.6 (±0.09) |
| Cameroun | 4.2 (±0.82) | 4.1 (±1.77) | 3.4 (±0.94) | 4.2 (±0.28) | 1.2 (±0.74) | 3.1 (±0.35) |
| South Africa | \| 4.7(±0.02) \| \| --- \| \|  \| | \| 4.2 (±0.01) \| \| --- \| \|  \| | \| 4.3 (±0.05) \| \| --- \| \|  \| | \| 3.2(±0.005) \| \| --- \| \|  \| | \| 3.5 (±0.048) \| \| --- \| \|  \| \|  \| | \| 2.9 (±0.12) \| \| --- \| \|  \| |

|  | *M. africanum* |  | *M. bovis* |  | *M. caprae* |  |
| --- | --- | --- | --- | --- | --- | --- |
|  | Influent | Effluent | Influent | Effluent | Influent | Effluent |
|  | Median (±SD) | Median (±SD) | Median (±SD) | Median (±SD) | Median (±SD) | Median (±SD) |
| Ghana | 3.8(±0.01) | 3.0 (±0.38) | 4.6 (±0.86) | 3.5 (±0.75) | 2.1 (±1.95) | 2.2 (±1.57) |
| Nigeria | 2.6 (±1.35) | 3.8 (±0.83) | 3.1 (±0.23) | 3.8 (±0.10) | 2.5 (±0.75) | 1.9 (±1.30) |
| Kenya | 3.4 (±0.79) | 1.9 (±0.75) | 2.9 (±0.32) | 1.8 (±0.75) | 3.5 (±1.81) | 1.7 (±0.75) |
| Uganda | 2.8 (±0.40) | 3.4 (±0.03) | 2.8 (±1.87) | 3.3 (±2.09) | 3.1 (±0.35) | 2.3 (±1.15) |
| Cameroun | 2.5 (±1.63) | 3.3 (±0.61) | 2.8 (±0.10) | 3.1 (±2.40) | 2.6 (±1.30) | 2.1 (±1.30) |
| South Africa | \| 3.2(±0.24) \| \| --- \| \|  \| | \| 2.8(±0.02) \| \| --- \| \|  \| | \| 3.3(±0.01) \| \| --- \| \|  \| | \| 3.1(±0.01) \| \| --- \| \|  \| | \| 3.8(±0.10) \| \| --- \| \|  \| | \| 2.8(±0.02) \| \| --- \| \|  \| |
|  |  |  |  |  |  |  |
